# Zoonotic Transmission of Tuberculosis Between Pastoralists and Their Livestock in South-East Ethiopia

**DOI:** 10.1007/s10393-012-0754-x

**Published:** 2012-04-17

**Authors:** Balako Gumi, Esther Schelling, Stefan Berg, Rebuma Firdessa, Girume Erenso, Wondale Mekonnen, Elena Hailu, Ermias Melese, Jemal Hussein, Abraham Aseffa, Jakob Zinsstag

**Affiliations:** 1 Jimma University College of Agriculture and Veterinary Medicine, P.O. Box 307, Jimma, Ethiopia; 2 Swiss Tropical and Public Health Institute, P.O. Box, 4002 Basel, Switzerland; 3 University of Basel, Basel, Switzerland; 4 Animal Health and Veterinary Laboratories Agency, New Haw, Surrey KT15 3NB UK; 5 Armauer Hansen Research Institute, P.O. Box 1005, Addis Ababa, Ethiopia

**Keywords:** mycobacteria, *Mycobacterium tuberculosis*, *Mycobacterium bovis*, humans, cattle, camels

## Abstract

Despite huge global efforts in tuberculosis (TB) control, pastoral areas remain under-investigated. During two years sputum and fine needle aspirate (FNA) specimens were collected from 260 Ethiopian pastoralists of Oromia and Somali Regional States with suspected pulmonary TB and from 32 cases with suspected TB lymphadenitis. In parallel, 207 suspected tuberculous lesions were collected from cattle, camels and goats at abattoirs. All specimens were processed and cultured for mycobacteria; samples with acid-fast stained bacilli (AFB) were further characterized by molecular methods including genus and deletion typing as well as spoligotyping. Non-tuberculous mycobacteria (NTM) were sequenced at the 16S rDNA locus. Culturing of AFB from human sputum and FNA samples gave a yield of 174 (67%) and 9 (28%) isolates, respectively. Molecular typing was performed on 173 of these isolates and 160 were confirmed as *Mycobacterium tuberculosis*, three as *M. bovis*, and the remaining 10 were typed as NTMs. Similarly, 48 AFB isolates (23%) yielded from tuberculous lesions of livestock, of which 39 were molecular typed, including 24 *M. bovis* and 4 NTMs from cattle, 1 *M. tuberculosis* and 1 NTM from camels and 9 NTMs from goats. Isolation of *M. bovis* from humans and *M. tuberculosis* from livestock suggests transmission between livestock and humans in the pastoral areas of South-East Ethiopia

## Introduction

Ethiopia ranks seventh among the world’s 22 countries with high tuberculosis (TB) disease burden and had an estimated incidence rate of 379 cases per 100,000 people per year (WHO 2008). *Mycobacterium tuberculosis* is the most common cause of human TB, but an unknown proportion of cases are due to *M. bovis*. TB caused by *M. bovis* (bovine tuberculosis; bTB) is clinically indistinguishable from TB caused by *M. tuberculosis* and can only be differentiated by laboratory methods (Cosivi et al. 1998). Specific data on zoonotic bTB transmission is very scarce in the developing world because the diagnosis of TB most often relays on sputum microscopy only. However, fairly recent molecular methods like spoligotyping (Kamerbeek et al. 1997) and deletion typing (Brosch et al. 2002) allow for identification of *M. bovis*.

Although cattle are considered to be the main hosts of *M. bovis*, isolations have been made from many other livestock and wildlife species and transmission to humans constitutes a public health problem (Ayele et al. 2004; OIE 2009). In many developing countries, bTB remains endemic causing significant economic losses (Zinsstag et al. 2006). In animals, bTB has been reported from 33 of 43 African countries (Ayele et al. 2004). Human cases of bTB have been described in Ghana, Niger, Uganda and Tanzania (Idigbe et al. 1986; Addo et al. 2007; Oloya et al. 2008) and in immigrants from Chad (Godreuil et al. 2010).

The proportion of bTB in human TB is estimated to be <5% worldwide (Cosivi et al. 1998; Michel et al. 2010). But this figure is based on estimates and we lack empirical representative data on the proportion of human *M. bovis* among all TB patients at national level. This information would be important for the estimation of the societal cost of bTB.

Routes of transmission to people are likely to be through consumption of untreated milk and meat products from infected animals, but also via aerosol in the proximity to livestock. These possible risk factors are of particular concern for many developing countries where pasteurization is limited and where people are living close to their animals.

In Ethiopia, several prevalence studies have been performed recently that show that bTB is endemic in cattle; however, prevalences vary depending on the geographical areas, breeds and husbandry practices. Abattoir and dairy farm studies from central Ethiopia have reported prevalence between 3.5 and 13.5% and locally in peri-urban Addis Ababa up to 50% (Ameni et al. 2007; Shitaye et al. 2007; Berg et al. 2009; Demelash et al. 2009; Regassa et al. 2010). In contrast, lower prevalence of 0.9% was reported in traditionally kept zebu cattle (Tschopp et al. 2010a). Other livestock than cattle have also been investigated. Based on gross pathology, prevalences of 5–10% were reported in camels slaughtered at Dire Dawa abattoir in eastern Ethiopia and in Addis Ababa abattoir (Mamo et al. 2009; Mamo et al. 2011). Hiko and Agga (2011) reported a 4.2% prevalence of bTB in goats slaughtered at the Mojo export abattoir in central Ethiopia. The observed variability of bTB disease frequency in Ethiopia might well be influenced by different livestock production systems (rural/pastoral/peri-urban) and different geographic and climatic contexts. Transmission of bTB seems to be higher in intensive peri-urban settings when compared to extensive rural and pastoral areas. Hence, a detailed understanding of bTB transmission requires field studies in a given social and ecological context.

Most of these studies focused on prevalence in cattle in the central highlands of Ethiopia. However, little data on human and animal TB is available from the major pastoralist areas in South-East Ethiopia, particularly from the Somali Region, which are often difficult to access also due to insecurity. Few studies have been conducted in southern Ethiopia. The abattoir bTB prevalence of Borana pastoralist cattle was 4% (Demelash et al. 2009), individual comparative intradermal tuberculin test prevalences were 0.8% in cattle of Hamer pastoralists in South Omo (Tschopp et al. 2010b) and 4.4% among Guji-Boran pastoralist cattle (Gumi et al. 2011). It appears that the prevalence of bTB in pastoral areas of Southern Ethiopia is relatively low. However, since pastoralists live in close proximity with their animals, animal-to-human transmission of bTB might still be significant. The potential of transmission of zoonotic TB in South-East Ethiopia was unknown. The objectives of this study were first to assess the presence of *M. bovis* among human TB patients and to describe mycobacterial strains circulating in South-East Ethiopian pastoralists and their livestock using a “One health” approach, studying human and livestock hosts simultaneously (Zinsstag et al. 2009). Second, data from this study should then be compared with the overall epidemiological situation in Ethiopia.

## Materials and Methods

### Study Area

**Figure 1:**
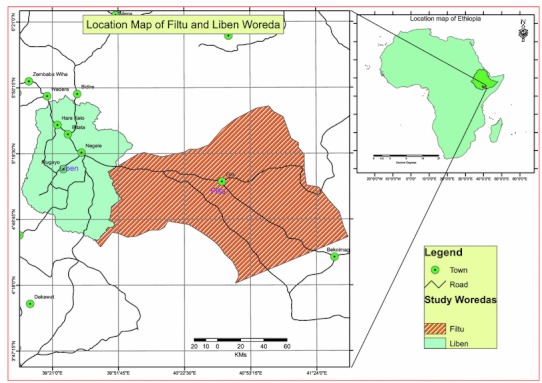
Location map of study areas (Liben and Filtu Woredas, South-East Ethiopia).

The study was conducted from March 2008 to February 2010 in South-East Ethiopia in the Guji (Negelle) and Liben (Filtu) zones of Oromia and Somali Regional States (Fig. 1). The lowland of the Guji zone is inhabited by pastoral and agro-pastoral communities whose livelihood is based on livestock production. The Liben (Filtu) zone of Somali region is arid lowland inhabited by pastoral communities. Study area has highest live stock population density in the country and is major source of livestock for the domestic and export markets. Cross-border movement of pastoral communities and their livestock to neighbouring countries (Kenya and Somalia) is common.

### Sample Collection

**Figure 2:**
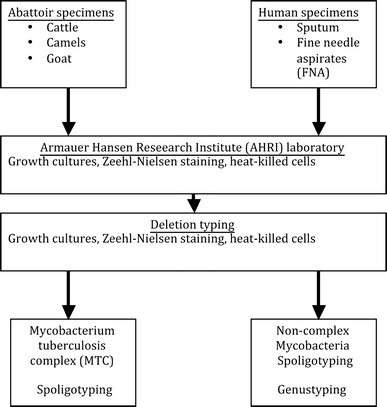
Laboratory processing flow chart for human (sputum and FNAs) and animal (lung and gut associated lymphnodes).

Ethical clearance for the study was obtained from the Ethics committee of Basel (Ref. 147/08, AHRI/ALERT; Ref. P010/08) and the Ethiopian National Ethical Review Committees (Ref. RDHE/65-86/2009). After informed consent was obtained from participants, sputum samples from suspected pulmonary TB patients and fine needle aspirates (FNAs) samples from suspected TB lymphadenitis patients were collected by trained laboratory technicians or physicians. FNA specimens were collected and stored in cryo-tubes with phosphate buffer saline (PBS) pH 7.2, and sputum specimens were collected in sterile containers. Suspected tuberculous lesions were collected by trained meat inspectors from cattle carcasses at Negelle abattoir and from camels and goats slaughtered at Filtu slaughterhouse. Similarly, sampling of suspected tuberculous lesions was also performed on camels and goats that were traceable back to the pastoral areas in South-East Ethiopia at Mojo and Addis Ababa abattoirs. All animal specimens were preserved in PBS in 30-ml plastic sterile universal containers. All human and animal specimens were stored at 4°C until transported on ice within 5 days to the Armauer Hansen Research Institute (AHRI) laboratory in Addis Ababa. In case of unavailable transportation means, samples were kept in the regions at −20°C before transport and further processing at AHRI. A flow chart of the sample collection, processing and molecular typing is provided in Fig. 2.

### Culturing and Molecular Typing

All specimens were processed according to standard methods. Tuberculous-like animal lesions were dissected and manually homogenized, then decontaminated with 4% NaOH for 15 min and centrifuged at 3,000 rpm for 15 min. The sediment was neutralized with 2 N HCl using phenol red as an indicator and inoculated on three different media slants: Two Löwenstein–Jensen (LJ) media supplemented either with glycerol or pyruvate, and Middlebrook 7H11 media (Berg et al. 2009). The slants were incubated at 37°C for 8 weeks and examined daily for the first week and then weekly for the presence of mycobacterial colonies. Cultures were considered negative if no visible growth was detected after 8 weeks of incubation. Microscopic examination of cultures using the Ziehl–Neelsen staining method was performed to detect the presence of acid-fast-positive bacilli (AFB) (Roberts et al. 1991). AFB-positive cultures were prepared as 20% glycerol stocks and stored at −80°C as reference.

Heat-killed cells of each AFB isolate were prepared by mixing ~2 loopful of cells (≥20 μl cell pellet) in 200 μl dH_2_O followed by incubation at 80°C for 1 h. Heat-killed AFB samples were used as templates in multiplex polymerase chain reactions (PCR) for typing of *Mycobacterium* genus and region of difference (RD; deletion typing), according to protocols previously described (Berg et al. 2009). Each isolate characterized as non-tuberculous mycobacteria (NTM) was sequenced at the 16S rDNA locus and the sequence was entered in the Basic Local Assignment Search Tool (BLAST) database at the National Center for Biotechnology Information (NCBI) and the Ribosomal Differentiation of Microorganisms (RIDOM) (http://rdna.ridom.de) database for further identification of species (Berg et al. 2009). DNA sequencing was performed at the Animal Health and Veterinary Laboratories Agency (AHVLA), United Kingdom, using an Applied Biosystems model 3730 automated capillary DNA sequencer. Isolates genetically identified by deletion typing as of the *M. tuberculosis* complex (MTC) were spoligotyped for further strain characterization as previously described (Kamerbeek et al. 1997). Spoligotyping data were compared with the Spoligo-International-Typing (SIT) database (http://www.pasteur-guadeloupe.fr:8081/SITVITDemo/ and http://www.cs.rpi.edu/~bennek/tbinsight/tblineage.html to match SIT numbers and lineage classifications. Isolates identified as *M. bovis* were compared with spoligotype patterns in the international *M. bovis* database (www.mbovis.org). Spoligotype patterns of all MTC isolates were analysed using spolTools (http://www.emi.unsw.edu.au/spolTools) (Tang et al. 2008).

### Molecular Typing Methods (Adapted From Müller 2008)

#### Spacer Oligonucleotide Typing (Spoligotyping)

Spoligotyping makes use of the variability of the MTC chromosomal direct repeat (DR) locus for strain differentiation (Kamerbeek et al. 1997). The DR region is composed of multiple well-conserved DRs of 37 bp which are separated by non-repetitive 34–41 bp spacer sequences. In the standard spoligotyping scheme, a PCR with primers complementary to the DR-sequence is used to amplify all spacer sequences of a given strain. One of the two primers is labelled with a biotin marker. The PCR products are denatured and hybridized to a standard set of 43 oligonucleotides covalently linked to a membrane. These oligonucleotides correspond to 37 spacers from *M. tuberculosis* H37Rv and 6 additional spacers from *M. bovis* BCG P3. If any of these spacers are also present in an investigated strain, they will be amplified during the PCR and hybridized to the spacers on the membrane. The successful hybridization can be visualized by incubation with streptavidin peroxidase (which binds to the biotin molecule), subsequent addition of a chemiluminescent streptavidin peroxidase substrate and exposure to a light sensitive film. The presence or the absence of each individual spacer sequence will generate a spoligotype pattern for the specific strain that was typed.

#### Large Sequence Polymorphism (LSP) Analysis

LSPs generally refer to large genomic deletions or insertions. Large genomic deletions (also called regions of difference, RD) are widely used for the phylogenetic analyses of MTC but are not appropriate for molecular epidemiological studies due to a low mutation rate (Gagneux and Small 2007). The genomic deletion RD9 discriminates *M. tuberculosis* from the other members of the MTC. If RD9 is intact, a strain is considered as *M. tuberculosis*. Similarly, *M. bovis* is deleted for RD4 while all other members of the MTC are not. Thereby, deletion typing of RD4 allows for identification of *M.* *bovis.*

## Results

### Sample Collection and Culturing Yield

A total of 292 patients clinically diagnosed with either pulmonary TB or TB lymphadenitis were recruited in Negelle and Filtu hospitals (Table 1). Sputum of 260 TB cases was cultured with a culturing yield of 164 (67%) AFB-positive isolates, while FNA samples were taken from 32 cases with TB lymphadenitis of which culturing yielded in nine (28%) AFB-positive isolates (Table 1). In parallel, 207 samples were collected from cattle, camels and goats with suspected TB lesions in Negelle, Filtu, Mojo and Addis Ababa abattoirs. Culturing yielded in 48 (23%) isolates that were identified as AFB-positive isolates (Table 2; Fig. 2).

**Table 1:** Numbers of Human Specimen that were Cultured and RD9 Deletion Typed from Sputum and FNAs from Negelle and Filtu Hospital

Specimen	Negelle Hospital	Filtu Hospital	AFB positive	Deletion typing	*M. tuberculosis*	*M. bovis*	NTM	Spol
Sputum	192	68	174	164	154	3	7	156
FNA	14	18	9	9	6	0	3	5
Total	206	86	183	173	160	3	10	161

AFB positive is the number of are acid-fast positive sputa and FNA samples.

RD9 deletion is the number of strains on which RD 9 was tested.

*M. tuberculosis* is the number of *M. tuberculosis* strains.

*M. bovis* is the number of *M. bovis* strains.

NTM is typed as *Mycobacterium* species not from the *M. tuberculosis* complex.

Spol is the isolates available for spoligotype analysis.

**Table 2:** Numbers of Abattoir Specimen that were Cultured and RD4 Deletion Typed from Negelle, Filtu, Addis Ababa and Mojo

Livestock species investigated (*N*)	Sites	Colleted and processed specimens	AFB positive	Deletion typing	*M. tuberculosis*	*M. bovis*	NTM	Spol
Cattle (5250)	Negelle	50	36	28	0	24	4	24
Camels (694)	Filtu = 181 Addis Ababa = 513	81	3	3	1	0	1	1
Goats (1744)	Filtu = 244 Mojo = 1500	76	9	9	0	0	9	0
Total		207	48	40	1	24	14	25

AFB positive is the number of are acid-fast positive sputa and FNA samples.

RD4 deletion is the number of strains on which RD 4 was tested.

*M. tuberculosis* is the number of *M. tuberculosis* strains.

*M. bovis* is the number of *M. bovis* strains.

NTM is typed as *Mycobacterium* species not from the *M. tuberculosis* complex.

Spol is the isolates available for spoligotype analysis.

*N* number of animals inspected.

### RD4 and RD9 Deletion Typing

To further characterize the AFB isolates, we used deletion typing to identify strains from the *M. tuberculosis* complex (MTC). Out of the 164 sputum isolates tested for RD9, 154 had intact RD9 locus and were subsequently classified as *M. tuberculosis*, while three isolates were RD9-deleted. The latter strains were also found to be deleted for RD4, a characteristic of *M. bovis*, and were declared as *M. bovis* strains. Assays of the remaining seven isolates did not generate any PCR product and were classified as NTM. Among the nine FNA isolates, six were *M. tuberculosis* (RD9 intact), whereas three isolates were NTM (Table 1).

**Fig. 3:**
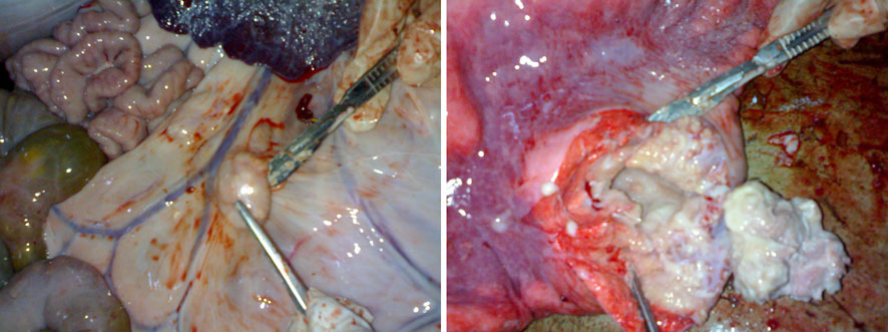
Granulomatous lesions from camel: enlarged mesenteric lymph node (*left*) and cross section of a caseous granulomatous lesion in the lung (*right*). The mycobacterium isolated from this lesion was characterized as *M. tuberculosis* (Photo: E. Meles).

A total of 40 livestock isolates were RD4 deletion typed, of which 28, 3 and 9 isolates were from cattle, camels and goats, respectively (Table 2). Out of 28 cattle isolates, 24 were *M. bovis* (RD4 deleted), while 4 were suggested as NTM. Among the three isolates from camels, one was characterized as *M. tuberculosis* from an animal with disseminated TB lesions (Fig. 3), one was suggested as an NTM and one requires further characterization. None of the nine AFB isolates from goats was typed as of MTC.

### Genus Typing and 16S rDNA Sequencing

Isolates that had failed to produce a PCR product in the deletion typing assays were tested by genus typing. In total, 24 isolates were NTM (Tables 1, 2) and six of these isolates were further identified by partial sequencing of the 16S rDNA gene. Three isolates were from goats were *M. terrae* complex strains, *M. arupense* and *Corynebacterium pseudotuberculosis*, while two isolates from cattle were both *M. fortuitum*. One of the ten human NTM isolates was characterized as *M. flavescens* (Table 3).

**Table 3:** Identified Non-complex Mycobacteria (NTM) Isolates from 16S rDNA Locus Sequencing Results

Bacterial species	Source
*Mycobacterium terrae* complex	1 Goat
*Mycobacterium arupense*	1 Goat
*Corynebacterium pseudotuberculosis*	1 Goat
*Mycobacterium fortuitum*	2 Cattle
*Mycobacterium flavescens*	1 Human sputum

### Spoligotyping of Human Isolates

A total of 161 strains from sputum and FNA samples were spoligotyped. Five lineages could be recognized in this strain collection based on spoligotype features characteristic for each lineage (Brudey et al. 2006; Comas et al. 2009). The Euro-American (E-A) and the Central-Asian (CAS) lineages were dominating with 73.3 and 17.4%, respectively, while 6.8% of the strains belonged to the East-African-Indian (EAI) lineage. One isolate (0.6%) had the Beijing spoligotype pattern and the three *M. bovis* strains (1.9%) were also recognized by their characteristic spoligotype feature with missing spacers 3, 9, 16 and 39–43 (Table 4). Strains characterized as *M. tuberculosis* by the deletion typing were represented by 48 different spoligotype patterns, of which 15 had not yet been registered in SpolDB4, the international spoligotyping database (Brudey et al. 2006). Among the unregistered patterns, eleven strains belonged to the E-A lineage, one to the CAS and one to the EAI lineage. Nineteen strains were clustered with an average cluster size of 3 and a clustering ratio of 0.32, while 31 strains had unique patterns. The largest cluster belonged to the spoligotype SIT 149 and accounted for 24% of the 161 isolates. Strains of this type lack spacers 10–19 and 33–36, and constitute a sublineage named T3-ETH (Brudey et al. 2006). Two other more common patterns were SIT37 of the E-A lineage and SIT25 of the CAS lineage. The three *M. bovis* strain isolated from sputum were of spoligotypes SB0133 and SB0303 in the Mbovis.org database.

**Table 4:** Spoligotypes of *M. tuberculosis* Complex Strains Isolated from Humans and Livestock in South-Eastern Ethiopia

* Lineages/sub-lineages as defined by Brudey et al. (2006) and Berg et al. (2011).

*SIT No* Spoligo-International-Typing as described in SpolDB4 (Brudey et al. 2006), *SB No* www.mbovis.org.

*SP* sputum, *FN* FNA, *Ct* cattle, *Cm* camel, *E-A* Euro-American, *CAS* Central-Asian, *EAI* East-African-Indian.

### Spoligotyping of Animal Isolates

A total of 25 MTC strains from livestock were characterized by spoligotyping. The 24 strains of *M. bovis* isolated from cattle were represented by six different spoligotype patterns, two clusters and four unique patterns. The average cluster size was 3.6 with a clustering ratio of 0.3.

The vast majority of livestock *M. bovis* isolates were of spoligotype SB0133, while the remaining strains showed a slightly diverting spoligotype patterns SB0933, SB1942 and SB1983 (Table 4). The latter two types were new to the database Mbovis.org. All *M. bovis* strains lacked spacers 3–7, 9, 16, and 39–43. The single *M. tuberculosis* strain isolated from camel had spoligotype SIT 149 of the E-A lineage, the in this study most commonly isolated *M. tuberculosis* type.

## Discussion

### Human Isolates

This study suggests strongly that transmission of the causative agents of TB occur between humans and livestock in the pastoralist settings of Ethiopia. Indeed, our most recent typing data shows the exact same spoligotype and 24-loci MIRU-VNTR type (data not shown) for a human sputum and a cattle *M. bovis* isolate from the study area. However, despite close contact between humans and livestock, including consumption of raw milk and meat by pastoral communities, the incidence rate and subsequent prevalence of *M. bovis* in human TB patients was lower than expected. Higher prevalences of *M. bovis* in human TB patients have been reported in Uganda (7%) (Oloya et al. 2008), Ghana (3%) (Addo et al. 2007) and in Nigeria (4 and 15%) (Idigbe et al. 1986; Mawak et al. 2006). This variation may be due to differences in transmission pathways or in sampling and diagnostic techniques, for example, Oloya et al. (2008) sampled from lymph node biopsy of cervical TB lymphadenitis instead of FNA. The former produces higher culture yields. However, a recent study from central Ethiopia identified no *M. bovis* in patients with TB lymphadenitis when sampling from lymph node biopsies were performed (Beyene et al. 2009).

Even if the true prevalence of TB due to *M. bovis* among the pastoralists in Ethiopia is not yet well understood, this study shows that a few percents of the pastoralists suffering from TB may in fact have bTB (in our study 2%). Therefore, in a country with a high TB burden as is the case with Ethiopia, the number of patients that may require treatment for bTB may become critical. It is interesting to notice that the three patients identified with bTB had pulmonary disease and it raises the question if transmission by aerosol rather than by ingestion of contaminated food products were the causes of infection. The observed low numbers of recruited TB lymphadenitis cases in this study may not be representative of the disease prevalence since the majority of pastoralists encountered in the study area were not aware of the possibility for diagnosis and treatment of TB lymphadenitis at health facilities.

*Mycobacterium tuberculosis* strains isolated in this study belonged to the E-A, CAS, EAI and Beijing lineages. Epidemiologically, the most important type within the E-A lineage was SIT 149 (T3-ETH). This strain is isolated frequently in Ethiopia and among Ethiopian immigrants in Denmark (SpolDB4, http://www.pasteur-guadeloupe.fr/tb/bd_myco.html; Brudey et al. 2006). The CAS lineage is primarily found in East Africa (including this study area; SpolDB4; Groenheit et al. 2011), North India and Pakistan, reflecting intercontinental human migration (Gagneux et al. 2006; Gagneux and Small 2007). Identification of strains of the EAI lineage among the pastoralists is interesting since this lineage has not yet been reported from the north and central Ethiopia. Its presence among the southern Ethiopian pastoralists may be explained by a closer contact with pastoral communities in Somalia and Kenya, where the EAI lineage has been described previously (SpolDB4; Brudey et al. 2006), rather than contacts of people from the northern and the central highlands of Ethiopia (Gutacker et al. 2006). Further investigation is required to determine the epidemiological significance of the Beijing strain noted in this study. In Africa, isolates of the Beijing lineage are most common in Southern Africa but occurs sporadically in East Africa as well (Groenheit et al. 2011). The *M. bovis* isolates from human pulmonary TB patients matched with both the dominant spoligotype of the animal isolates in the area (SB0133) and with SB0303, which has been isolated from cattle in central Ethiopia and in other countries of East Africa (Berg et al. 2011), thus indicating cattle-to-human transmission. All *M. bovis* isolates collected in this study had spacers 3–7 missing, a spoligotype feature that serves as a marker for strains belonging to the African 2 lineage of *M. bovis* that is highly prevalent in East Africa (Berg et al. 2011). It is therefore likely that the *M. bovis* isolates of this study belong to the African 2 lineage but further typing is needed for final categorization.

### Animal Isolates

The most common spoligotype pattern among the animal isolates was SB0133. Previously this spoligotype was reported as the second most dominant strain in Ethiopian cattle and it is a common type in East Africa (Berg et al. 2011; Biffa et al. 2010). The clustering rate of 0.3 of *M. bovis* in cattle found in this study, related to the observed prevalence of intradermal tuberculin test of 4.4% among Guji–Boran pastoralist cattle (Gumi et al. 2011), indicates ongoing endemic stable transmission of a dominant strain. Single isolates belonged to SB1942 and SB1983, which are new spoligotypes, whereas SB0933 was previously reported from France (Haddad et al. 2001). None of the isolates from goats could be identified as of the MTC. Other authors reported isolation of *M. bovis* and *M. tuberculosis* from goats slaughtered at the Mojo export abattoir in Ethiopia; however, their diagnosis were based only on colony morphology and discrimination by culture on growth media with pyruvate or glycerol (Hiko and Agga 2011). The low yield from gross lesions specimens in livestock is consistent with existing data from Ethiopia (Berg et al. 2009). This may be due to variable diagnostic capacity of meat inspectors or because of the presence of other granulomatous diseases in livestock.

The *M. tuberculosis* strain isolated from disseminated TB lesions in a camel belongs to the E-A lineage (SIT 149), a dominant strain in Ethiopia (Brudey et al. 2006). This is the first known report of *M. tuberculosis* from a camel in Ethiopia, indicating likely human to camel transmission. Isolation of *M. tuberculosis* from gross TB lesions was recently reported in Nigerian goats (Cadmus 2009) and is more frequently found in Ethiopian cattle (Berg et al. 2009; Ameni et al. 2010). The close contacts between pastoralist communities and their livestock may be conducive for human to animal *M. tuberculosis* transmission, but further investigation is needed to determine the public health significance. We consider the finding of *M. tuberculosis* in camel as a rare event. In Ethiopia, *M. tuberculosis* seems to be more frequently transmitted from humans to livestock than *M. bovis* from cattle to humans.

### Non-tuberculous Mycobacteria

Approximately 10% of the AFB-positive isolates were characterized as NTM by molecular typing. Environmental mycobacteria are known to be opportunistic pathogens in HIV patients, but limited information is available for the bacterial isolates in this study. Previously reported were *M. fortuitum* in humans and livestock (Diguimbaye-Djaïbe et al. 2006; Mawak et al. 2006; Berg et al. 2009; Tschopp et al. 2010a, b) and *M. flavescens* and *M. terrae* complex in wildlife in South-West Ethiopia (Tschopp et al. 2010a, b). The presence of *M. fortuitum* could indicate the presence of farcy in Ethiopia as it is difficult to distinguish it from *M. farcinogenes* (Diguimbaye-Djaïbe et al. 2006). Its isolation from both animals and humans merits further investigation.

### Epidemiology of *M. bovis* in South-Eastern Ethiopia

The parallel study on the prevalence of bTB in pastoral cattle herds in the Oromia region, southern Ethiopia (Gumi et al. 2011) indicated a true prevalence below 10%, which is in the range of endemically stable transmission in sedentary rural areas of Ethiopia (Tschopp et al. 2010a), but slightly higher to the Hamer area (Tschopp et al. 2010b). The transmission dynamics of sedentary rural and mobile pastoral cattle is likely similar, with early exposure of calves, a variable latency period and transmission from adult cows through the airways and udder. This could explain the low level endemic transmission with a high herd prevalence. Compared to sedentary rural communities, where we could not find human *M. bovis* (publication in preparation), cattle–human transmission seems effective in pastoral communities in South-Eastern Ethiopia. Hence there is likely a higher exposure of pastoralists to *M. bovis*, when compared to sedentary communities.

## Conclusion

Identical spoligotypes of *M. bovis* isolates from humans and cattle, as well as collection of *M. tuberculosis* isolates from animals, indicates transmission between livestock, mainly between cattle and humans. Therefore, TB is of public health importance in pastoral settings of South-East Ethiopia and warrants locally adapted diagnosis and treatment protocols. *M. bovis* is naturally resistant to pyrazinamide, a commonly used treatment for TB. TB programs in areas where *M. bovis* is a potential etiologic agent in humans should therefore not neglect the zoonotic risk of bTB. *M. tuberculosis* isolates were represented by diversified lineages, requiring further typing to establish their position in the global TB population structure. This simultaneous study of mycobacteria in humans and livestock allowed relating transmission risks. It demonstrates an added value of a “One Health” approach of closer cooperation of human and animal health sectors.
